# Effect of bacterial load versus duration of exposure to bacteria on plasma TNFα concentrations in porcine fecal peritonitis

**DOI:** 10.1186/cc9668

**Published:** 2011-03-11

**Authors:** T Correa, L Brander, S Djafarzadeh, R Schröder, J Takala, A Reintam Blaser, M Vuda, S Mathias Jakob

**Affiliations:** 1 University Hospital Bern - Inselspital and University of Bern, Switzerland

## Introduction

The clinical relevance of preclinical sepsis research has been questioned [1]. This may in part be the result of varying degrees of experimental inflammatory insults. The objective of this study was to quantify inflammation based on plasma TNFα levels after exposure to two different bacterial loads, and after different lengths of bacterial incubation in the peritoneal cavity.

## Methods

We retrospectively evaluated plasma TNFα concentrations measured before and 24 hours after fecal peritonitis induced by 1 g/kg autologous feces (16 anesthetized pigs, median weight: 40.0 kg) and after 6, 12 and 24 hours of fecal peritonitis induced with 2 g/kg autologous feces (24 anesthetized pigs (*n *= 8/group); median weight: 41.0 kg). All animals were resuscitated with fluids, and antibiotics, and were mechanically ventilated according to standardized protocols. Differences along time after fecal peritonitis induced with 2 g/kg feces were assessed by ANOVA for repeated measures. Comparison between the two models (1 g/kg vs. 2 g/kg) after 24 hours of peritonitis was performed with an independent *t *test.

## Results

**Figure 1:**
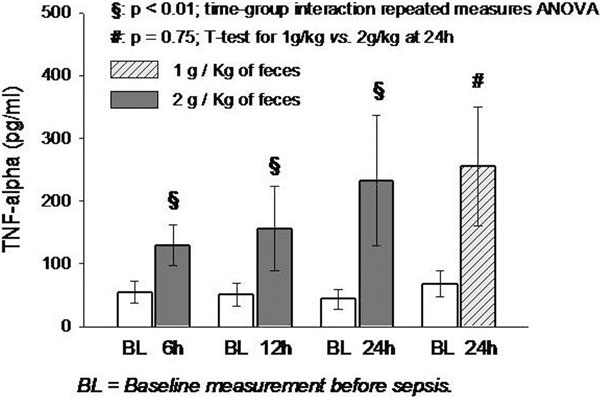
**Mean (95% CI) TNFα levels**.

TNFα increased from baseline to 6, 12 and 24 hours of peritonitis induced with 2 g/kg feces (*P *< 0.001 for time-group interaction) (Figure 1). The mean (± SD) plasma TNFα levels measured 24 hours after fecal peritonitis induced with 1 and 2 g/kg were 255 ± 178 pg/ml and 233 ± 124, respectively (*P *= 0.75; 95% CI for the difference: -124 to 169 pg/ml).

## Conclusions

The magnitude of inflammation expressed as plasma TNFα concentrations was associated with the duration of bacterial incubation in the peritoneal cavity but not with the amount of bacterial load. This has implications for the interpretation of experimental sepsis findings.
